# Rational Use of Antibiotics and Education Improved *Aeromonas* Necrotizing Fasciitis Outcomes in Taiwan: A 19-Year Experience

**DOI:** 10.3390/antibiotics11121782

**Published:** 2022-12-08

**Authors:** Tsung-Yu Huang, Yao-Hung Tsai, Ching-Yu Lee, Wei-Hsiu Hsu, Cheng-Ting Hsiao, Yao-Kuang Huang, Yen-Yao Li, Jiun-Liang Chen, Shu-Fang Kuo, Jo-Chun Hsiao, Hsing-Jung Li, Chien-Hui Hung, Kuo-Ti Peng

**Affiliations:** 1 Division of Infectious Diseases, Department of Internal Medicine, Chang Gung Memorial Hospital, Chiayi 61363, Taiwan; 2 College of Medicine, Chang Gung University, Taoyuan 33303, Taiwan; 3 Microbiology Research and Treatment Center, Chang Gung Memorial Hospital, Chiayi 61363, Taiwan; 4 Department of Orthopedic Surgery, Chang Gung Memorial Hospital, Chiayi 61363, Taiwan; 5 Department of Orthopedics, Taipei Medical University Hospital, Taipei 11031, Taiwan; 6 Department of Orthopaedics, School of Medicine, College of Medicine, Taipei Medical University, Taipei 11031, Taiwan; 7 Department of Emergency Medicine, Chang Gung Memorial Hospital, Chiayi 61363, Taiwan; 8 Division of Thoracic and Cardiovascular Surgery, Chiayi Chang Gung Memorial Hospital, Chiayi 61363, Taiwan; 9 Department of Laboratory Medicine, Chang Gung Memorial Hospital, Chiayi 61363, Taiwan; 10 Department of Medical Biotechnology and Laboratory Sciences, College of Medicine, Chang Gung University, Taoyuan 33303, Taiwan; 11 Department of Plastic and Reconstructive Surgery, Chang Gung Memorial Hospital, Chiayi 61363, Taiwan; 12 Department of Pediatrics, St. Martin De Porres Hospital, Chiayi 60069, Taiwan; 13 Graduate Institute of Clinical Medical Sciences, College of Medicine, Chang-Gung University, Taoyuan 33302, Taiwan

**Keywords:** necrotizing fasciitis, *Aeromonas*, rational antibiotic usage, infectious disease physician, education

## Abstract

Background. *Aeromonas* necrotizing fasciitis (NF) causes high rates of amputation and mortality, even after aggressive surgical debridement and antibacterial therapy. This study investigated the effects of rational use of antibiotics and education by infectious disease (ID) physicians on *Aeromonas* NF treatment outcomes. Methods. Retrospective review for conducted for four years (period I, without an ID physician, December 2001 to December 2005) and 15 years (period II, with an ID physician, January 2006 to March 2021). In period II, the hospital-wide computerized antimicrobial approval system (HCAAS) was also implemented. A pretest-posttest time series analysis compared the two periods. Differences in clinical outcomes, demographics, comorbidities, signs and symptoms, laboratory findings, *Aeromonas* antibiotic susceptibility, and antibiotic regimens were compared between the two periods. Results. There were 19 patients in period I and 53 patients in period II. Patients had a lower rate of amputation or mortality in period II (35.8%) compared with period I (63.2%). Forty-four patients (61.1%) had polymicrobial infections. In the emergency room, the rate of misdiagnosis decreased from 47.4% in period I to 28.3% in period II, while effective empiric antibiotic usage increased from 21.1% in period I to 66.0% in period II. After the ID physician’s adjustment, 69.4% received monotherapy in period II compared to 33.3% in period I. Conclusions. Because *Aeromonas* NF had a high mortality rate and was often polymicrobial, choosing an antibiotic regimen was difficult. Using the HCAAS by an experienced ID physician can improve rational antibiotic usage and clinical outcomes in *Aeromonas* NF.

## 1. Introduction

In 1952, Wilson used necrotizing fasciitis (NF) to describe the infection’s most consistent characteristic, fascial necrosis [1]. When NF is present, the underlying muscle is intact while the skin appears normal. Upon histopathology, swelling, necrosis, and inflammation were evident in the skin, skin fat, and fascia. There is a prominent presence of thrombosis at all levels of vessels [2].

*Aeromonas* NF is a rare and life-threatening necrotizing skin and soft tissue infection (NSSTI) characterized by rapidly spreading necrosis in the subcutaneous layers, especially in the fascia [1,3]. Despite aggressive surgical debridement and antibacterial treatment, *Aeromonas* NF causes high rates of amputation and mortality, with 27.3–50% of patients losing limbs [4,5,6] and 26.7–100% dying [4,5,6,7,8].

**Figure 1:**
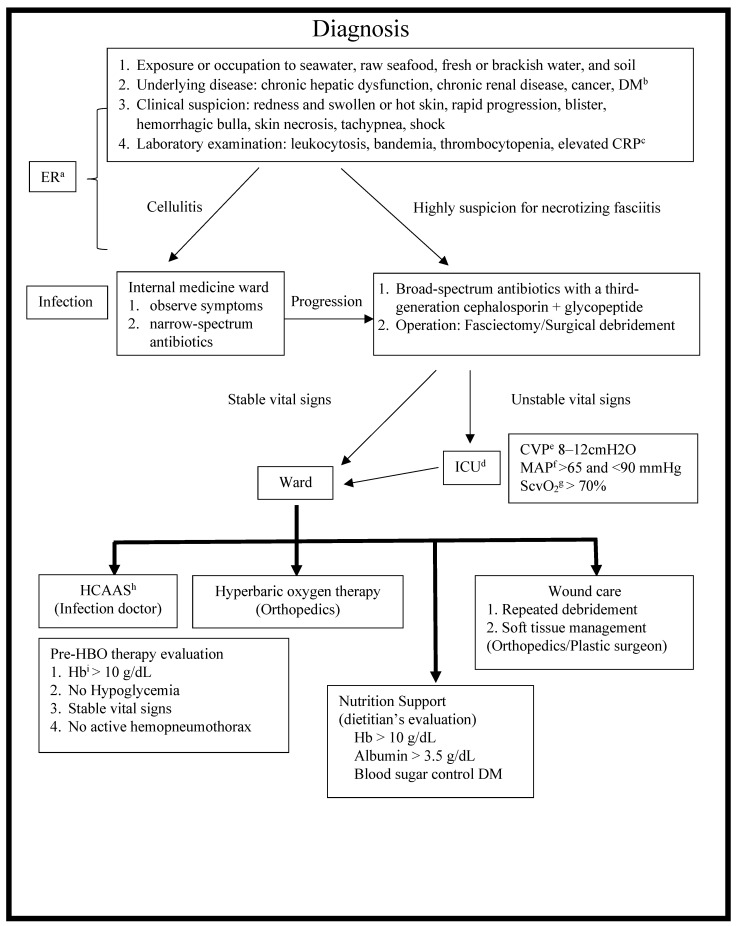
Diagnosis and treatment protocol of NF. NF, necrotizing fasciitis; ^a^ ER, emergency room; ^b^ DM, diabetes mellitus; ^c^ CRP, C-reactive protein; ^d^ ICU, intensive care unit; ^e^ CVP, central venous pressure; ^f^ MAP, mean arterial pressure; ^g^ ScvO_2_, central venous oxygen saturation; ^h^ HCAAS, hospital-wide computerized antimicrobial approval system; ^i^ Hb, hemoglobin.

The Chang Gung Memorial Hospital (CGMH)-Chiayi is a tertiary teaching hospital located on the western coast of southern Taiwan, and it has been in service since December 2001. Residents in this region are typically exposed to seawater, raw seafood, brackish water, and soil. As a result, there has been a relatively high incidence of *Vibrio* spp. and *Aeromonas* spp. infections in our hospital [4,5,6,7,8,9,10,11,12]. We set up a *Vibrio* NSSTIs Treatment and Research (VTR) Group comprising professional staff from various departments, including emergency medicine, orthopedic surgery, infectious disease (ID), plastic surgery, and the intensive care unit (ICU), and the hyperbaric oxygen treatment center [6,13,14]. The VTR Group also set up an NF diagnosis and treatment protocol (Figure 1).

The hospital-wide computerized antimicrobial approval system (HCAAS) is an online antimicrobial control system implemented at the CGMH in Taiwan [15,16,17]. The HCAAS reduces antimicrobial consumption and expenditures without compromising or reducing healthcare quality [15,16,17]. By using HCAAS and education by ID physicians, we evaluated *Aeromonas* NF treatment outcomes and improved rational antibiotic use.

## 2. Methods

### 2.1. Study Design

A retrospective study was conducted by the VTR Group at CGMH-Chiayi from December 2001 to March 2021. The study enrolled patients admitted to the emergency room (ER) with *Aeromonas* NF of the limbs. A pretest-posttest time series analysis compared the two study periods: period I (without an ID physician, December 2001 to December 2005 [4 years]) and period II (with an ID physician, January 2006 to March 2021 [15 years]). Data such as clinical outcomes, demographics, comorbidities, presenting signs and symptoms, laboratory findings, *Aeromonas* antibiotic susceptibility, and antibiotic regimens were recorded and compared between the two periods. This study protocol was approved by the Institutional Review Board of Chang Gung Medical Foundation (Number: 202001656B0, 201801530B1B0, 97-1073B, 99-1123C, and 102-5105B), and all patients provided informed consent.

### 2.2. HCAAS

**Figure 2:**
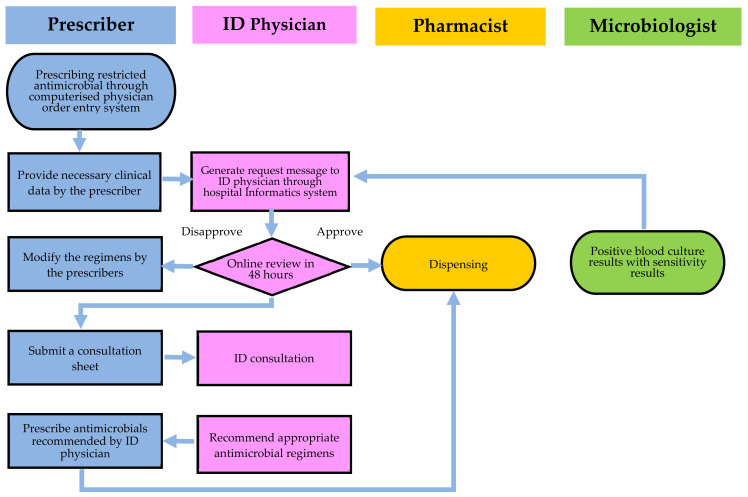
An overview of the hospital-wide computerized antimicrobial approval system (HCAAS). Infectious diseases (ID) physicians have three options following online review of the antimicrobial prescription: approval, disapproval, or an on-site consultation.

A HCAAS application is an intranet-based system that integrates with electronic medical records [14,15,16]. The entire algorithm is displayed in Figure 2 of the system flow. In the ICU, all parenteral antimicrobial agents must be approved by ID physicians. ID physicians are notified when antimicrobial agents are prescribed in preassigned regions. The information above is automatically processed by HCAAS. The ID physician will review online patient clinical records, laboratory reports, cultures, and images submitted by the prescribing physician. The ID physician consults with the physician in charge before making a decision. When a disapproval decision is made, the unit-dose delivery system of the pharmacy will discontinue the antimicrobial after 48 h, and the prescriber will be notified immediately.

### 2.3. Definitions

For inclusion in the *Aeromonas* NF patient group, the following criteria were used: (1) necrosis of the skin, subcutaneous fat, superficial fascia, or muscles beneath the skin; (2) *Aeromonas* sp. isolated from soft-tissue lesions and/or blood collected during surgery or after patients arrived at the ER [17,18].

The following antibiotics are not restricted: cefazolin, penicillin G, oxacillin, ampicillin, gentamicin, clindamycin, metronidazole, and trimethoprim-sulfamethoxazole. Many other antibiotics are restricted, such as cephalosporins (cefuroxime, ceftriaxone, ceftazidime, cefpirome, and cefepime), flomoxef, penicillins (amoxicillin, amoxicillin/clavulanate, ampicillin/sulbactam, piperacillin, and piperacillin-tazobactam), carbapenems (imipenem, meropenem, doripenem, and ertapenem), aztreonam, amikacin, fluoroquinolones (ciprofloxacin, levofloxacin, and moxifloxacin), glycopeptides (vancomycin and teicoplanin), and other kinds of antibiotics (colistin, tigecycline, linezolid, and daptomycin).

Antimicrobial regimens prescribed before the results of the microbiologic susceptibility test became available were defined as empirical, while those subsequently adjusted according to the microbiologic susceptibility test results were defined as definitive [18]. Effective empirical antimicrobial usage was defined as the administration of an initial antimicrobial regimen that provided coverage for all infectious isolates based on antimicrobial susceptibility testing [11,19].

### 2.4. Laboratory Methods

Aeromonas species consists of oxidase-positive, polar flagellated, glucose-fermenting, facultatively anaerobic, motile bacteria that do not grow in gram-negative rods containing 6.5% NaCl. Conventional methods were used to identify all strains. For further verification, API-20E and ID32 GN Systems (bioMérieux Inc., Hazelwood, MO, USA) or Vitek 2 ID-GNB identification cards (bioMérieux Inc., Durham, NC, USA) were used. Clinical and Laboratory Standards Institute (CLSI) criteria for microorganisms were used to interpret the results of antimicrobial susceptibility tests.

### 2.5. The Judgment of Antimicrobial Use and Education by ID Physicians

Through the HCAAS and consultation formally, ID physicians find all NF patients and follow up on these patients’ conditions. ID physicians reviewed the initial empirical antimicrobial prescriptions, categories of judgment for antibiotic regimen at ER, and definitive antibiotic regimen (≥72 h after admission). ID physicians recorded all inappropriate prescriptions during admission and recommendations on medical charts and further classified them by escalation, de-escalation, and other reasons [17,19]. ID physicians have been conducting combined meetings with emergency medicine doctors and educating the diagnosis of NF, rational antimicrobial usage, microbial resistance, and infection control every 2 months since March 2008 till now.

### 2.6. End-Point Measurements

This study was conducted to compare the outcome of treatment of *Aeromonas* NF between a new, large hospital in the two different periods with and without ID physicians, which had not been previously studied. The endpoints were mortality, amputation rate, effective rates of empirical antimicrobial usage at ER, and definitive antibiotic regimen after admission.

### 2.7. Statistical Analysis

Fisher’s exact test was used to test continuous variables and Student’s *t*-tests were used to test categorical variables. When the *p*-value was less than 0.05 with a two-tailed test, it was considered statistically significant. To compare gradients between periods I and II, segmented linear regression techniques were used. Statistics were calculated using SPSS 25.0 (SPSS Corporation, Chicago, IL, USA).

## 3. Results

### 3.1. Uptake

From December 2001 to March 2021, 72 patients admitted via the ER were surgically confirmed to have *Aeromonas* NF of the limbs. Period I had 19 admissions and period II had 53. Table 1 lists the demographic data, characteristics, and clinical outcomes.

### 3.2. Inpatient Outcomes

**Figure 3:**
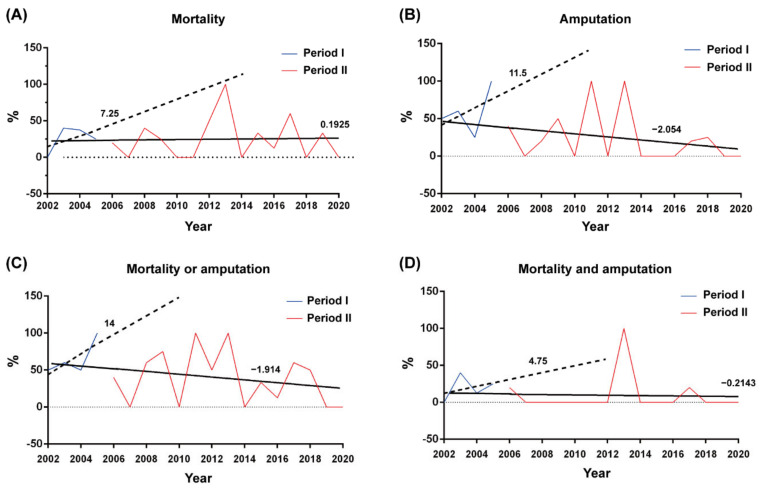
The hospitalized patients, the rates of (**A**) mortality, (**B**) amputation, (**C**) mortality or amputation, and (**D**) mortality and amputation per year before (blue lines) and after (red lines) the deployment of the HCAAS in January 2006. The trend lines established by linear regression before and after deployment of HCAAS are depicted as dotted and solid lines, respectively.

Period II had a lower rate of amputation (17.0%) than period I (52.6%), in addition to a lower rate of amputations or mortality (35.8%) than period I (63.2%). The trend lines are listed in Figure 3. The 95% confidence interval was listed in Table 2. The rate of misdiagnosis in the ER decreased from 47.4% in period I to 28.3% in period II, and a delay of >24 h in taking the patient from the ER to surgery decreased from 42.1% in period I to 26.4% in period II, without statistical difference.

### 3.3. Demographic Data

Period II was characterized by a statistically higher Charlson score (6.1 ± 3.1) than period I (4.5 ± 2.7), in addition to a higher incidence of chronic liver dysfunction but a lower rate of peripheral vascular disease (Table 1).

### 3.4. Antimicrobial Resistance and the Empiric Antimicrobial Prescription

The proportion of bloodstream infection was higher in period II than in period I, without a statistical difference (Table 3). We found a higher susceptibility rate of *Aeromonas* to penicillin in period II (81.1%) than period I (26.3%), with a statistically significant difference.

The effective empirical and appropriate antimicrobial usage in the ER increased from 21.1% in period I to 66.0% in period II and from 15.8% in period I to 32.1% in period II, respectively. Empirical antimicrobial regimen escalation decreased from 78.9% in period I to 30.2% in period II, but de-escalation increased from 5.3% in period I to 30.2% in period II. After admission >72 h later, 15 patients (78.9%) in period I and 45 patients (84.9%) in period II were changed to different antimicrobial regimens. In period II, more antimicrobial regimens were re-adjusted to monotherapy (69.4%) than in period I (33.3%).

### 3.5. Microbiological Analysis

*A. hydrophila* was the most common infectious bacterium, accounting for 49 of 72 patients (68.1%), followed by 10 patients with *A. sobria* (13.9%), 10 with *Aeromonas* sp. (13.9%), and 3 with *A. caviae* (4.2%). Of the 72 patients, 44 (61.1%) were diagnosed with polymicrobial infections. The most common isolates obtained from patients with co-infective microorganisms were *Clostridium* sp. (21, 47.7%), followed by *Enterobacter* sp. (14, 31.8%) (Table 4).

### 3.6. Clinical Presentation

There were no significant differences in the presentation of fever (>38 ℃), tachycardia (heartbeat > 100/min), tachypnea (respiratory rate > 20/min), or shock (systolic blood pressure < 90 mmHg) and in the proportion of patients presenting with erythematous, swollen, painful lesions; bullae formation; and skin necrosis between the two periods (Table 5).

### 3.7. Laboratory Findings

More than 10% of banded leukocyte cells were observed more frequently in period II than in period I (Table 5). In addition, the serum albumin level was lower (*p* = 0.005) in period II than in period I.

### 3.8. Surgical Treatment

When the first operation was performed in period I, three patients (15.8%) underwent amputation, five (26.3%) underwent debridement, and eleven (57.9%) underwent fasciotomy. In period II, the first surgery performed was amputation for two patients (3.8%), debridement for 12 (22.6%), and fasciotomy for 39 (73.6%).

## 4. Discussion

Aeromonads are flagellated, rod-shaped, nonsporulating facultative anaerobic gram-negative bacteria of the family *Aeromonadaceae* with typing of the aquatic environment [20]. Currently, there are more than 20 *Aeromonas* species identified, of which three, namely *A. hydrophila*, *A. caviae*, and *A. veronii* biovar *sobria*, are the major clinical important [20]. These bacteria can cause human infections, including hepatobiliary infections, blood-borne infections, traumatic events, and burns and scalds related to skin and soft-tissue infections, in previously healthy subjects [6,18,21,22,23].

This 19-year study is the first report on an online antimicrobial control system used by ID specialists for *Aeromonas* NF treatment. A delay of >24 h in the first surgical intervention from symptom onset to surgery adversely affects survival outcomes [3,24]. This delay of >24 h decreased from 42.1% in period I to 26.4% in period II. Mortality is associated with a delay in the diagnosis of NF [25]. In the ER, the mean rate of misdiagnosis is 71.4% (range from 41% to 96%) [26], while in this study, the rate of misdiagnosis was only 27.8% in period II. Due to prompt NF diagnosis, surgery can be arranged as quickly as possible. The trend lines indicate that mortality or amputations decreased between periods II and I (Figure 3). We consider persistent education of emergency medicine doctors by an ID physician should help in improving this delay [27]. In period I, we aggressively amputated patients’ limbs to save their lives, but we still found higher mortality rates than in period II. In the first operation, we change the patient’s surgical treatment from amputation to fasciotomy. In this manner, period II has lower amputation rates and rates of mortality or amputations.

With more than 19 years of experience in NF treatment, our VTR Group found a lot of medical information, clinical phenomena, and important laboratory findings for the early diagnosis of NF, especially that caused by *Vibrio* and *Aeromonas* spp. Because *Aeromonas* spp. are often located in fresh or brackish water, sewage, or nonfecal organic materials [7,22]. First, we need to take the occupational history of farmers, recent trauma, and exposure to soil, wood, or dirty ditches. Second, a significantly higher mortality rate was observed in patients with NF who also had chronic kidney disease, chronic liver dysfunction, diabetes mellitus, or cancer [5,6,24,28,29]. About 55–57% of patients with *Aeromonas* NF had chronic liver dysfunction [5,14]. Hepatic cirrhosis was found in 36–54% of patients with *Aeromonas* bacteremia [18,22] and in 27–32% of patients with *Aeromonas* NF [5,6]. So, we aggressively checked viral hepatitis and liver cirrhosis in period II.

Third, *Aeromonas* NF was often initially present with tachypnea, shock, hemorrhagic bullae, and skin necrosis [4,5,6,7,14]. These important early clinical signs and symptoms are easily observed when patients arrive at the ER. Patients with *Aeromonas* NF have a statistical tendency to have tachypnea and initially present with septic shock tending to mortality [6]. The emergence of hemorrhagic bullae is considered a feature of *Vibrio* infection, but they are also found in 38–40% of patients with *Aeromonas* NF [6,14]. Patients with NF presenting with hemorrhagic bullae have a higher rate of amputation and mortality than patients with serous-filled bullae or without bullae [14]. In one study, 27.9% of patients with *Aeromonas* NF had skin necrosis, and this phenomenon is a poor predictor of mortality [6]. In period II, more patients had tachypnea, shock, and skin necrosis, but there was no statistically significant difference between period I and period II.

Fourth, we also found some important laboratory results to predict the poor outcome of NF. Lower counts of total and segmented leukocytes, higher counts of banded leukocytes, thrombocytopenia, and lower serum albumin levels are significantly associated with mortality [6,8,30,31]. More than 10% of these forms of leukocytes indicate gram-negative NF, especially that caused by *Vibrio* spp. and *Aeromonas* spp. [6,8,30]. In this study, we also found more patients with >10% banded leukocytes and lower serum albumin levels in period II.

The fatality rate associated with *Aeromonas* bacteremia is reported to be 30–36% [18,22]. In our previous study, 38.2% of patients with *Aeromonas* NF had bacteremia [6], and bloodstream infection significantly increased the mortality rate in NF [6,28]. A total of 17–61% of patients with NF receive postoperative intubation [14,30], and 36–100% of patients require ICU care [6,14,25,30]. Although patients exhibited high disease severity in period II, including a higher Acute Physiology and Chronic Health Evaluation II score, a higher bacteremia rate, postoperative intubation, and ICU care, lower rates of mortalities or amputations were found. These better outcomes must rely on the long-term cooperation of VTR Group.

At ER, empiric antibiotic therapy with oxacillin plus gentamicin was prescribed for suspicion of NF in period I, and third-generation cephalosporins were empirically prescribed when a *Vibrio* infection was suspected in period II [30]. Antibiotics ordered with evidence of culture and susceptibility results are found to be more appropriate than those ordered empirically [32]. *Aeromonas* SSTIs are often polymicrobial [6,21] and have high drug resistance [6], so the selection of an antimicrobial regimen is challenging. The initial ineffective empirical antimicrobial usage is related to poor outcomes for patients with *Aeromonas* NF [6].

However, culture-directed antimicrobial therapy and an antibiotic order with consultation with an ID physician for critically ill patients are more likely to be appropriate [7,17,32]. The HCAAS and persisted on-the-spot education by ID physicians in a trauma ICU can decrease 54% sepsis-related and 41% overall infection-related mortality rates, respectively, and increase the appropriate ratio of antibiotic usage from 60.5% to 73.7% [17]. Early prompt fasciotomy, combined with appropriate empiric antimicrobial therapy supported by ID physicians and aggressive ICU care, should be initially administered to critically ill patients suffering from fulminant NF to save lives and limbs [7,17,24,33].

Most *Aeromonas* strains are uniformly resistant to penicillin but invariably susceptible to aminoglycosides, sulfa drugs, second–fourth-generation cephalosporins, carbapenems, fluoroquinolones, and tetracyclines [22,34,35]. Third-generation cephalosporins combined with tetracycline are commonly the empiric prescription in highly suspected *Vibrio* infection cases before the pathogen is identified [36,37]. But the high resistance of cephalosporins was related to *Aeromonas* NF mortality [6]. Fluoroquinolones are seemed higher active against *Aeromonas* spp. [6,35], but a mutation in the *gyrA* gene induces fluoroquinolone resistance [35]. However, in highly suspected fulminate *Aeromonas* NF cases, we sometimes add quinolones before the final pathogen is identified [6]. Most *Aeromonas* spp. infections are treatable with monotherapy and studies with combination therapy do not show better outcomes [38]. In period II, 84.9% of patients adjusted the definitive antibiotic regimen based on the ID physician’s suggestion using the HCAAS, monotherapy was more common, simpler, and rational than combination therapy. At the same time, we should limit antibiotic prescriptions to patients, and we should reduce the length of time these treatments are required [39].

The HCAAS provided a comprehensive evaluation of a novel, ID specialist-led, and sustainable system to provide antibiotic stewardship in a busy medical ecology [17]. HCAAS was not used in this study to reduce antimicrobial consumption, expenditure, and de-escalation, thus helping ID physicians detect such major diseases earlier. The average duration of hospitalization for survivors is usually more than 30 days [6,14,24,30]. ID physicians can monitor the following antibiotic regimen through the HCAAS during the working day and suggest an appropriate antibiotic regimen to improve rational antibiotic usage.

The major limitation of this study was that the HCAAS is only used at admission but not in ER. Second, there was no report of the minimum inhibitory concentration of *Aeromonas* spp. Third, we did not analyze antibiotic consumption, the occurrence of new *Aeromonas* antibiotic-resistant strains, and the rates of hospital-associated infection.

## 5. Conclusions

As soon as possible, long-term, close cooperation and experienced teamwork can help diagnose and aggressively treat *Aeromonas* NF. An effective antimicrobial regimen against *Aeromonas* spp. confirmed by an experienced ID physician using the HCAAS can improve clinical outcomes and rational antibiotic usage. However, antibiotic resistance is also an important consideration.

## Figures and Tables

**Table 1:** Demographic data, characteristics, and clinical outcomes of 72 patients with *Aeromonas* NF ^a^ between periods I and II.

Variable	Period I (*n* = 19)	Period II (*n* = 53)	*p*-Value
Age (years)	61.4 ± 14.6	61.9 ± 14.9	0.896
Gender, male	14 (73.7)	44 (83.0)	0.378
Disease severity			
APACHE ^b^ II score	13.0 ± 6.9	16.3 ± 8.9	0.149
Postoperative intubation	7 (36.8)	28 (52.8)	0.232
Need ICU ^c^ care	9 (47.4)	34 (64.2)	0.201
Charlson score	4.5 ± 2.7	6.1 ± 3.1	0.043 *
Underlying chronic diseases			
Chronic kidney disease	5 (26.3)	24 (45.3)	0.148
Chronic liver dysfunction	6 (31.6)	37 (69.8)	0.004 *
Viral hepatitis	6 (31.6)	27 (50.9)	0.146
Liver cirrhosis	3 (15.8)	21 (39.6)	0.059
Cancer	5 (26.3)	14 (26.4)	0.993
Diabetes Mellitus	11 (57.9)	27 (50.9)	0.603
Peripheral vascular disease	6 (31.6)	4 (7.5)	0.009 *
Misdiagnosis at ER ^d^	9 (47.4)	15 (28.3)	0.130
ER to surgery > 24 h	8 (42.1)	14 (26.4)	0.221
ER to surgery (h)	46.7 ± 83.3	32.1 ± 65.0	0.442
Clinical outcomes			
No. of mortalities	6 (31.6)	13 (24.5)	0.550
No. of amputations	10 (52.6)	9 (17.0)	0.002 *
No. of mortalities or amputations	12 (63.2)	19 (35.8)	0.039 *
No. of mortalities and amputations	4 (21.1)	3 (5.7)	0.052
No. of debridement	3.2 ± 2.5	3.1 ± 2.0	0.913
ICU stay (days)	4.0 ± 6.7	9.4 ± 18.8	0.226
Hospital stays (days)	26.5 ± 20.6	36.5 ± 29.4	0.176

Data were presented as the mean (standard deviation) or frequency (%). * *p* < 0.05. ^a^ NF, necrotizing fasciitis; ^b^ APACHE, Acute Physiology and Chronic Health Evaluation; ^c^ ICU, intensive care unit; ^d^ ER, emergency room.

**Table 2:** A 95% confidence interval for the rates of mortality, amputation, mortality or amputation, and mortality and amputation between periods I and II.

		95% Confidence Intervals	
		Low	High
Mortality	period I	−29.81	44.31
	period II	−3.69	4.075
Amputation	period I	−53.15	76.15
	period II	−6.574	2.467
Amputation or mortality	period I	−22.51	50.51
	period II	−6.606	2.778
Amputation and mortality	period I	−32.93	42.43
	period II	−3.7	3.272

**Table 3:** Microbiological results and judgment for antimicrobial agents for 72 patients with *Aeromonas* NF between periods I and II.

Variable	Period I (*n* = 19)	Period II (*n* = 53)	*p*-Value
Bloodstream infection	5 (26.3)	21 (39.6)	0.300
Polymicrobial infection	10 (52.6)	34 (64.2)	0.377
Susceptibility (Disk diffusion method)			
Aminoglycosides	15 (78.9)	46 (86.8)	0.415
Carbapenems	13 (68.4)	33 (62.3)	0.632
Cephalosporins	14 (73.7)	42 (79.2)	0.617
Fluoroquinolones	19 (100)	51 (96.2)	0.390
Penicillin	5 (26.3)	43 (81.1)	<0.001 *
Sulfa drugs	16 (84.2)	47 (88.7)	0.613
Tetracycline	19 (100)	46 (86.8)	0.095
Effective empirical antimicrobial usage at ER	4 (21.1)	35 (66.0)	0.001 *
Categories of judgment for antibiotic regimen			0.003 *
Appropriate	3 (15.8)	17 (32.1)	
Dis-appropriate			
Escalation	15 (78.9)	16 (30.2)	
De-escalation	1 (5.3)	16 (30.2)	
Other reasons	0 (0)	4 (7.5)	
Definitive antibiotic regimen (≥72 h after admission)	*n* = 15	*n* = 49	
Monotherapy	5 (33.3)	34 (69.4)	0.012 *
Cephalosporins	5 (33.3)	32 (65.3)	
Fluoroquinolones	0 (0)	2 (4.1)	
Combination therapy	10 (66.7)	15 (30.6)	0.012 *
Cephalosporins + metronidazole	2 (13.3)	8 (16.3)	
Cephalosporins + glycopeptide	1 (6.7)	2 (4.1)	
Cephalosporins + metronidazole + glycopeptide	1 (6.7)	1 (2.0)	
Penicillin + aminoglycosides	6 (40)	0 (0)	
Penicillin + metronidazole + monobactam	1 (6.7)	0 (0)	
Fluoroquinolones + metronidazole	0 (0)	2 (4.1)	
Fluoroquinolones + cephalosporins	0 (0)	2 (4.1)	

Data were presented as frequency (%). * *p* < 0.05.

**Table 4:** Summary of identified infectious microorganisms in 44 patients of *Aeromonas* polymicrobial NF of the limbs.

Identified Infectious Microorganisms	Total (%)
Gram-negative aerobic pathogens	
*Enterobacter* spp.	14 (31.8)
*Enterobacter cloacae*	13 (29.5)
*Enterobacter aerogenes*	1 (2.3)
*Klebsiella* spp.	11 (25.0)
*Klebsiella pneumoniae*	8 (18.2)
*Klebsiella oxytoca*	4 (9.1)
*Pseudomonas aeruginosa*	10 (22.7)
*Escherichia coli*	11 (25.0)
*Proteus vulgaris*	4 (9.1)
*Citrobacter freundii*	3 (6.8)
*Serratia marcescens*	2 (4.5)
*Vibrio vulnificus*	1 (2.3)
*Morganella morganii*	1 (2.3)
*Acinetobacter baumannii*	1 (2.3)
Gram-positive aerobic pathogens	
*Enterococcus* spp.	9 (20.5)
*Enterococcus faecalis*	8 (18.2)
*Enterococcus casseliflavus*	1 (2.3)
*Staphylococcus* spp.	4 (9.1)
MRSA ^a^	2 (4.5)
MSSA ^b^	2 (4.5)
Group D *Streptococcus*	1 (2.3)
*Anaerobic pathogens*	
*Clostridium* spp.	21 (47.7)
*Clostridium bifermentans*	9 (20.5)
*Clostridium* sp.	6 (13.6)
*Clostridium perfringens*	3 (6.8)
*Clostridium bifermentans*	1 (2.3)
*Clostridium butyricum*	1 (2.3)
*Clostridium novyi A*	1 (2.3)
*Peptostreptococcus* spp.	7 (15.9)
*Peptostreptococcus anaerobius*	4 (9.1)
*Peptostreptococcus* sp.	2 (4.5)
*Peptostreptococcus magnus*	1 (2.3)
*Bacteroides fragilis*	3 (6.8)
*Prevotella* sp.	3 (6.8)
Total	44 (100)

^a^ MRSA, methicillin-resistant *Staphylococcus aureus*; ^b^ MSSA, methicillin-sensitive *Staphylococcus aureus*.

**Table 5:** Comparison of clinical presentations and important laboratory findings of 72 patients with *Aeromonas* NF between periods I and II.

Variable	Period I (*n* = 19)	Period II (*n* = 53)	*p*-Value
Exposure to soil, wood, or dirty ditches	8 (42.1)	31 (58.5)	0.219
The duration of symptoms/signs (days)	3.5 ± 4.1	2.9 ± 2.9	0.515
Systemic symptoms/signs			
Fever (>38 ℃)	5 (26.3)	18 (34.0)	0.540
Tachycardia ^a^	11 (57.9)	31 (58.5)	0.964
Tachypnea ^b^	7 (36.8)	28 (52.8)	0.232
Shock ^c^	6 (31.6)	23 (43.4)	0.368
Limb’s symptoms/signs			
Local pain or tenderness	19 (100)	52 (98.1)	0.547
Local swelling	19 (100)	50 (94.3)	0.289
Hemorrhagic bullae	7 (36.8)	20 (37.7)	0.945
Serous bullae	1 (5.3)	6 (11.3)	0.444
Skin necrosis	4 (21.1)	16 (30.2)	0.446
Leukocytosis (WBC ^d^ count ≥ 12,000/uL)	8 (42.1)	27 (50.9)	0.508
Band forms (>10%)	1 (5.3)	15 (28.3)	0.038 *
Hemoglobin < 10 g/dL	7 (36.8)	17 (32.1)	0.705
Thrombocytopenia (platelet count <15 × 10^4^/uL)	9 (47.4)	30 (56.6)	0.488
eGFR ^e^ <30 c.c./min	4 (21.1)	19 (35.8)	0.235
Albumin (mg/dL)	2.7 ± 0.8	1.9 ± 0.8	0.005 *
Total bilirubin (mg/dL)	3.2 ± 3.6	4.5 ± 4.9	0.324
C-reactive protein (mg/dL)	133.3 ± 140.4	106.7 ± 103.8	0.445

Data were presented as the mean (standard deviation) or frequency (%). ^a^ Tachycardia: heart beat > 100/min. ^b^ Tachypnea: respiratory rate > 20/min. ^c^ Shock: systolic blood pressure < 90 mmHg; ^d^ WBC, white blood cell; ^e^ eGFR, estimat. * *p* < 0.05.

## Data Availability

Not applicable.

## Abbreviations

NF, necrotizing fasciitis; HCAAS, hospital-wide computerized antimicrobial approval system; ID, infectious disease; ER, emergency room; ICU, intensive care unit; NSSTI, necrotizing skin and soft-tissue infection; OR, odds ratio; CI, confidence interval.
